# Distinct Determinants of Hospitalisation and In-Hospital Mortality After Acute Stroke: Insights into Stroke–Heart Syndrome (SHS-PARTICLES Study)

**DOI:** 10.3390/jcm15197343

**Published:** 2026-09-22

**Authors:** Małgorzata Chlabicz, Magdalena Krętowska, Paweł Muszyński, Jan Kochanowicz, Sławomir Dobrzycki, Gregory Y. H. Lip, Łukasz Kuźma

**Affiliations:** 1 Department of Invasive Cardiology, Internal Medicine with CICU and Laboratory of Hemodynamics, Medical University of Bialystok, 15-089 Bialystok, Poland; 2 Department of Cardiology, Lipidology and Internal Medicine with Intensive Coronary Care Unit, Medical University of Bialystok, 15-089 Bialystok, Poland; 3 Department of Neurology with Stroke Unit, Medical University of Bialystok, 15-089 Bialystok, Poland; 4 Institute of Life Course and Medical Sciences, University of Liverpool, Liverpool L69 7ZX, UK; 5 Department of Clinical Medicine, Aalborg University, 9220 Aalborg, Denmark; 6 Department of Cardiac Surgery and Transplantology, National Medical Institute of the Ministry of Interior and Administration, 02-507 Warsaw, Poland

**Keywords:** stroke–heart syndrome, brain–heart axis, NT-proBNP, stroke

## Abstract

**Background/Objectives**: Stroke–heart syndrome (SHS) involves myocardial injury, cardiac dysfunction, and arrhythmias after acute stroke, playing a key role in prognosis. Most evidence comes from acute ischaemic stroke (IS), while cardiac involvement after haemorrhagic stroke (HS) is less understood. Additionally, the prognostic roles of cardiac biomarkers, neurological severity, and cardiac systolic function on early outcomes are not well defined. **Methods**: We retrospectively analysed 586 patients with stroke, assessing admission and peak high-sensitivity cardiac troponin I (hs-cTnI) and N-terminal pro-B-type natriuretic peptide (NT-proBNP), left ventricular ejection fraction (LVEF), length of hospital stay (LOS), and in-hospital mortality. Univariable and multivariable regression analyses evaluated factors associated with mortality and LOS. **Results**: The cohort comprised 586 patients with acute cerebrovascular events, including 560 (95.6%) with acute ischaemic cerebrovascular disease (ischaemic ACVD) and 26 (4.4%) with HS. HS patients had higher hs-cTnI elevations and concentrations, whereas NT-proBNP did not differ between stroke subtypes. In univariable analyses, age, hs-cTnI, NT-proBNP, LVEF, National Institutes of Health Stroke Scale (NIHSS), and modified Rankin Scale (mRS) were associated with in-hospital mortality. In multivariable analysis, peak NT-proBNP remained independently associated with mortality even after logarithmic transformation. Older age, higher NIHSS and mRS scores, and lower LVEF were associated with longer LOS only in univariable analyses. **Conclusions**: Cardiac and neurological abnormalities provide complementary prognostic roles after acute stroke. HS was associated with a more pronounced myocardial injury profile, whereas peak NT-proBNP was independently associated with in-hospital mortality. These findings support integrated neurological and cardiovascular risk assessment after acute stroke.

## 1. Introduction

Cardiovascular disease (CVD) remains the leading cause of morbidity and mortality worldwide, and stroke represents one of its most severe clinical manifestations [1]. Stroke remains the second leading cause of cardiovascular (CV) mortality and a major contributor to long-term disability worldwide [2]. In 2021, approximately 11.9 million new stroke cases occurred worldwide, and the global burden is expected to increase further with population ageing and the increasing prevalence of CV risk factors [3,4,5]. Ischaemic stroke (IS) and cardiovascular diseases (CVDs) share multiple underlying risk factors and pathophysiological pathways, including hypertension (HT), atrial fibrillation (AF), atherosclerosis, diabetes mellitus (DM), chronic kidney disease (CKD), and heart failure (HF) [6,7,8]. Importantly, the interaction between the CV and cerebrovascular systems is bidirectional: pre-existing cardiac disease may predispose to stroke, while acute cerebral injury may itself induce secondary cardiac dysfunction. This latter phenomenon is increasingly conceptualised as stroke–heart syndrome (SHS), encompassing acute myocardial injury, cardiac dysfunction, electrocardiographic abnormalities, arrhythmias, and acute coronary syndromes (ACSs) occurring after stroke [9,10]. Increasing evidence indicates that acute stroke may induce secondary myocardial injury, through complex neurohumoral mechanisms and disturbances of autonomic regulation within the brain–heart axis. Cardiac manifestations occur predominantly during the early phase after stroke, with the greatest burden observed within the first 72 h, although cardiovascular complications may develop throughout the subsequent weeks [11]. The occurrence of SHS is associated with an increased burden of adverse CV events, excess in-hospital mortality, and impaired functional recovery after stroke [12,13,14]. However, cardiac complications may be frequently missed or not properly identified at an early stage, partly because they can be masked by stroke-related symptoms [15].

Autonomic imbalance, sympathetic activation, systemic inflammation, catecholamine-mediated myocardial injury, and disruption of the central autonomic network have been proposed as central mechanisms linking cerebral injury with cardiac dysfunction. Recent work by Stengl, Scheitz et al. further emphasises that myocardial injury following stroke is heterogeneous and may reflect several overlapping mechanisms, rather than acute coronary plaque rupture alone [16]. Cardiac troponins and N-terminal pro-B-type natriuretic peptide (NT-proBNP) are increasingly used to characterise cardiac involvement after stroke. Elevated high-sensitivity cardiac troponin identifies myocardial injury and is consistently associated with adverse clinical outcomes, but its interpretation after acute stroke is challenging because myocardial injury may arise from ACS, demand ischaemia, pre-existing structural heart disease, renal dysfunction, or neurogenic mechanisms associated with SHS. Current evidence therefore supports assessing troponin within a broader clinical and CV context, rather than treating it as an isolated marker of myocardial infarction (MI) [17]. Importantly, circulating cardiac biomarkers may also reflect pre-existing subclinical CV dysfunction. In a recent study of individuals without acute illness, resting hs-cTnT and NT-proBNP concentrations were independently associated with indices of subclinical CV dysfunction [18].

Despite its clinical significance, no clearly defined diagnostic criteria, standardised screening strategies, or management guidelines currently exist for patients with SHS. Therefore, understanding how often SHS occurs, which factors contribute to its development, what mechanisms are involved, and which patients are most vulnerable remains an important unmet clinical need that is essential for developing effective prevention and treatment strategies. Moreover, most studies evaluating myocardial injury and neurocardiac biomarkers have focused on acute IS, whereas evidence on haemorrhagic stroke (HS) remains comparatively limited. Furthermore, the relative contributions of cardiac biomarker elevation, neurological injury severity, and underlying cardiac dysfunction to short-term outcomes, including mortality and duration of hospitalisation, remain incompletely understood. Recent studies of intracerebral haemorrhage nevertheless suggest that acute and dynamic myocardial injuries are also clinically relevant in this population, supporting the need for evaluation of SHS across stroke subtypes [19]. This study aimed to compare cardiac biomarker profiles and in-hospital outcomes between patients with HS and those with ACVD. It also sought to identify factors influencing in-hospital mortality and length of hospital stay. We hypothesised that the prognostic significance of myocardial injury, haemodynamic cardiac stress, neurological severity, and systolic cardiac function would vary by clinical outcome.

## 2. Materials and Methods

### 2.1. Study Population

The present study is a retrospective analysis of the SHS-PARTICLES cohort and included consecutive adult patients hospitalised for acute cerebrovascular events at the University Clinical Hospital in Białystok, Poland, in 2025. Acute cerebrovascular events comprised IS, transient ischaemic attack (TIA), and HS. Diagnosis was established according to contemporary clinical guidelines based on clinical assessment and neuroimaging.

Patients admitted within 72 h of symptom onset, with available admission high-sensitivity cardiac troponin I (hs-cTnI) and/or N-terminal pro-B-type natriuretic peptide (NT-proBNP) measurements, were eligible. We excluded patients with >30% missing clinical data.

We retrospectively extracted demographic characteristics, CV and cerebrovascular history, comorbidities, pharmacotherapy, neuroimaging findings, reperfusion treatment, laboratory measurements, electrocardiographic monitoring, echocardiographic findings, neurological status, and in-hospital outcomes from electronic medical records.

The study was conducted in accordance with the Declaration of Helsinki and was approved by the Bioethics Committee of the Medical University of Białystok (approval no. APK.002.53.2026; approval date: 19 February 2026). The requirement for individual informed consent was waived because of the retrospective design and use of anonymised, routinely collected clinical data.

### 2.2. Laboratory Measurements

Blood samples were obtained as part of routine clinical care. Cardiac biomarker assessment included hs-cTnI and NT-proBNP. Admission values were defined as the first available measurement following hospital admission, while peak concentrations represented the highest recorded value during hospitalisation. The analysis was performed using the Abbott Alinity analyser (Dallas (Irving), TX, USA) via chemiluminescent microparticle immunoassays (CMIA; Abbott Laboratories, Wiesbaden, Germany).

Elevated hs-cTnI was defined as >15.6 ng/L, corresponding to the upper reference limit used in the study, while marked troponin elevation was defined as >10 times the upper reference limit (>156 ng/L).

### 2.3. Neurological and Vascular Assessment

Neurological impairment was assessed using the National Institutes of Health Stroke Scale (NIHSS), while functional status was evaluated using the modified Rankin Scale (mRS). IS aetiology was classified according to the Trial of Org 10172 in Acute Stroke Treatment (TOAST) classification.

Carotid artery atherosclerosis was assessed by ultrasonography, performed as part of routine diagnostic evaluation during hospitalisation. Carotid artery stenosis was defined as ≥50% stenosis of the common and/or internal carotid artery on ultrasonography according to the North American Symptomatic Carotid Endarterectomy Trial (NASCET) criteria and hemodynamic assessment, based on peak systolic and end diastolic velocities, or ≥50% stenosis on CT angiography.

### 2.4. Echocardiographic Assessment

Transthoracic echocardiography was performed during hospitalisation as part of routine clinical assessment. Left ventricular systolic function was assessed using left ventricular ejection fraction (LVEF). LVEF was assessed using the biplane Simpson method by an experienced cardiologist, in accordance with the recommendations of the European Association of Cardiovascular Imaging (EACVI) and the American Society of Echocardiography (ASE).

### 2.5. In-Hospital Clinical Outcomes

The primary clinical outcome was in-hospital mortality. Secondary outcomes included LOS and a composite in-hospital clinical endpoint comprising MI, marked hs-cTnI elevation (>10 times the upper reference limit; >156 ng/L), or in-hospital death. All outcomes were restricted to the index hospitalisation; no post-discharge outcomes were included in the present analysis.

### 2.6. Statistical Analysis

We assessed the distribution of continuous variables using the Kolmogorov–Smirnov test and visual inspection of histograms. Normally distributed continuous variables are presented as mean ± standard deviation (SD), whereas non-normally distributed variables are presented as median with interquartile range (IQR). Categorical variables are presented as numbers and percentages.

Between-group comparisons were performed using Student’s *t*-test for normally distributed continuous variables and the Mann–Whitney U test for non-normally distributed continuous variables. Categorical variables were compared using the chi-square test.

We performed univariable logistic regression to evaluate factors associated with in-hospital mortality. Results are reported as odds ratios (ORs) with 95% confidence intervals (CIs). For analyses using biomarker concentrations on their original scale, ORs for hs-cTnI and NT-proBNP were expressed per 1000 ng/L and per 1000 pg/mL increase, respectively. We then used multivariable logistic regression to identify factors independently associated with in-hospital mortality. The multivariable models included age, history of heart failure (HF), peak hs-cTnI, peak NT-proBNP, and LVEF, selected a priori based on their established clinical relevance. Given the right-skewed distributions of hs-cTnI and NT-proBNP, two multivariable models were evaluated: Model 1 included peak biomarker concentrations on their original scale, whereas Model 2 included natural logarithm-transformed peak hs-cTnI and peak NT-proBNP concentrations.

We also assessed the discriminative ability of peak hs-cTnI and peak NT-proBNP for in-hospital mortality using receiver operating characteristic (ROC) curve analysis, and quantified it with the area under the ROC curve (AUC).

Factors associated with LOS were evaluated using univariable and multivariable linear regression analyses, with regression coefficients (β), standard errors (SEs), 95% CIs, and *p*-values reported. We evaluated two multivariable models: Model 1 included age, peak hs-cTnI, peak NT-proBNP, and LVEF, with biomarker concentrations on their original scale, whereas Model 2 included age, natural logarithm-transformed peak hs-cTnI and peak NT-proBNP, and LVEF.

Statistical analyses were performed using Statistica 13 (TIBCO Software Inc., Palo Alto, CA, USA) and Stata 18.0 (StataCorp LLC, College Station, TX, USA). All statistical tests were two-sided, and a *p*-value ≤ 0.05 was considered statistically significant.

## 3. Results

The study cohort comprised 586 patients with acute cerebrovascular events, with a median age of 75 (IQR 67–84); 47.61% were male. Of these, 560 patients (95.6%) had acute ischemic cerebrovascular disease (ischemic ACVD), including IS and TIA, and 26 patients (4.4%) had HS. Median age did not differ significantly between the HS and ischaemic ACVD groups (73 [66–78] vs. 75 [67–85] years, respectively; *p* = 0.18). We observed no significant disparities in sex distribution or most baseline comorbidities. AF was present in 33.96% of the overall cohort and did not differ significantly between the study groups. Carotid atherosclerosis documented via ultrasonography was less common in the HS cohort than in the ischemic ACVD cohort (61.5% vs. 81.25%; *p* = 0.01). The prevalence of previous CV and cerebrovascular events was comparable between the study groups. No significant differences were observed in the history of previous MI (7.69% in HS vs. 7.68%; *p* = 1.00), previous IS (3.85% vs. 12.68%; *p* = 0.18), or previous TIA (0.00% vs. 3.75%; *p* = 0.32). Table 1 presents baseline characteristics of the study population.

Elevated hs-cTnI (>15.6 ng/L) was present in 44.5% of the overall study population, while hs-cTnI >10× the upper reference limit (URL; 156 ng/L) occurred in 12.6% of patients. Elevated hs-cTnI was more frequent in patients with HS compared to those with ischemic ACVD (73.1% vs. 44.1%; *p* = 0.004). Patients with HS also had higher baseline median hs-cTnI concentrations at admission (21.1 [10–33.6] vs. 10 [10–27.1] ng/L; *p* = 0.03) and higher peak hs-cTnI levels (29.8 [12.7–53.0] vs. 12.5 [10–42.5] ng/L; *p* = 0.02) (Table 1). However, the prevalence of hs-cTnI >10 × URL did not differ significantly between the groups (19.2% vs. 12.3%; *p* = 0.3). Overall in-hospital mortality was 7.5%.

### Clinical Course, Length of Stay, and In-Hospital Mortality

Median LOS was significantly longer in patients with HS than in those with ischaemic ACVD (16.5 [4–20] vs. 8 [5–9] days; *p* < 0.001), whereas in-hospital mortality did not differ significantly between the groups (3.9% vs. 7.7%; *p* = 0.47).

In univariable logistic regression analysis of the entire cohort, older age, higher admission and peak hs-cTnI concentrations, higher admission and peak NT-proBNP concentrations, and higher NIHSS and mRS scores were associated with increased odds of in-hospital mortality, whereas higher LVEF was associated with lower odds of death (Table 2).

In multivariable logistic regression analysis using biomarker concentrations on their original scale (Model 1), peak NT-proBNP remained independently associated with in-hospital mortality (OR 1.114 per 1000 pg/mL increase, 95% CI 1.032–1.203; *p* = 0.006), whereas age, history of HF, peak hs-cTnI, and LVEF were not independently associated with mortality.

In Model 2, using natural logarithm-transformed peak hs-cTnI and peak NT-proBNP concentrations, ln-transformed peak NT-proBNP remained independently associated with in-hospital mortality (OR 1.866, 95% CI 1.008–3.452; *p* = 0.05), whereas ln-transformed peak hs-cTnI was not significantly associated with mortality (OR 1.236, 95% CI 0.912–1.674; *p* = 0.17) (Table 2).

Receiver operating characteristic (ROC) curve analysis of peak biomarker concentrations demonstrated an AUC of 0.774 for peak hs-cTnI and 0.742 for peak NT-proBNP for in-hospital mortality. The corresponding ROC curves are presented in Supplementary Figure S1.

In univariable linear regression analysis, longer LOS was associated with older age (β = 0.038; *p* = 0.020), higher NIHSS score (β = 0.096; *p* = 0.050), higher mRS score (β = 0.600; *p* = 0.004), and lower LVEF (β = −0.116; *p* < 0.001). Neither admission nor peak hs-cTnI or NT-proBNP concentrations were significantly associated with LOS (all *p* > 0.05) (Table 2).

In multivariable linear regression analysis, using biomarker concentrations on their original scale (Model 1), none of the included variables remained significantly associated with LOS, although lower LVEF showed a borderline association with longer hospitalisation (β = −0.094; *p* = 0.056). Similarly, in Model 2, which used natural logarithm-transformed peak hs-cTnI and peak NT-proBNP concentrations, none of the included variables was significantly associated with LOS (Table 2).

## 4. Discussion

In this study, we evaluated the relative contributions of cardiac biomarkers, neurological severity, and left ventricular systolic function to short-term outcomes after acute cerebrovascular events. Three main observations emerged. First, peak NT-proBNP was independently associated with in-hospital mortality, whereas hs-cTnI was not independently associated after multivariable adjustment. Second, patients with HS had a more pronounced myocardial injury profile, reflected by a higher prevalence of elevated hs-cTnI and higher admission and peak hs-cTnI concentrations than those with ischaemic ACVD. Third, factors associated with prolonged hospitalisation differed from those associated with mortality: neurological severity, functional impairment, older age, and reduced LVEF were associated with LOS in univariable analyses, but none remained significant in the multivariable models. Collectively, these findings support the concept that neurological and cardiac abnormalities provide complementary prognostic information after acute stroke.

Among the cardiac biomarkers assessed, NT-proBNP showed the most consistent association with in-hospital mortality. Both admission and peak NT-proBNP were associated with mortality in univariable analyses, and peak NT-proBNP remained independently associated with mortality after multivariable adjustment. NT-proBNP reflects myocardial wall stress and haemodynamic burden, and its circulating concentrations are also influenced by underlying cardiovascular disease, age, atrial fibrillation, and renal function [20]. Thus, elevated NT-proBNP during acute stroke may reflect not only the acute brain–heart response, but also the underlying individual CV vulnerability. Previous studies have similarly demonstrated increased NT-proBNP concentrations after acute stroke and associations with adverse outcomes [21]. Supporting our findings, peak NT-proBNP has also been identified as an independent risk factor for myocardial injury in acute stroke [22]. More recently, a systematic review and meta-analysis of 11 prospective observational studies demonstrated that NT-proBNP has prognostic value for mortality following IS [23]. The independent association of peak NT-proBNP with mortality observed in our cohort further supports prospective evaluation of serial NT-proBNP measurements as part of dynamic CV risk stratification after acute stroke. However, given the retrospective and non-standardised nature of biomarker sampling in our study, prospective studies with predefined sampling intervals are required before serial NT-proBNP assessment can be recommended for routine clinical practice.

However, further research is required in SHS to evaluate myocardial injury, particularly in specific patient groups. Indeed, a recent retrospective cohort study demonstrated that peak NT-proBNP, alongside coronary artery disease (CAD), insular cortex lesions, C-reactive protein (CRP), and a higher NIHSS score, were independent risk factors for myocardial injury in acute IS patients with DM [22]. The study by Prandin et al. found DM to be a risk factor for SHS, and that reperfusion treatment was associated with a lower rate of SHS in patients with severe anterior stroke [24]. These observations are further supported by a large population-based cohort study demonstrating that the 10-year cumulative incidence of HF was 13.5% among stroke patients, with HT, obesity, DM, and CKD identified as significant contributory risk factors [25]. These findings collectively highlight that comorbidity profiles substantially shape the risk of cardiac complications after stroke. Therefore, tailored management strategies are needed across stroke and cardiovascular disease subtypes, and a recent European Society of Cardiology (ESC) Council on Stroke position paper advocates a holistic, integrated care approach for these high-risk patients. [26].

In contrast to NT-proBNP, hs-cTnI was associated with mortality in univariable analyses, but did not retain independent prognostic significance in the multivariable model. This finding does not diminish the clinical relevance of troponin elevation after stroke. Cardiac troponin is a highly sensitive marker of myocardial injury, but elevated concentrations in acute stroke are aetiologically heterogeneous and may result from type 1 MI, type 2 MI, demand ischaemia, chronic structural heart disease, renal dysfunction, or neurogenic myocardial injury within the spectrum of SHS. This distinction is clinically important, as Nolte et al. showed that substantially elevated hs-cTn concentrations were associated with type 1 MI in patients with acute IS [27]. Our findings are also broadly consistent with research by Stengl et al., in which acute myocardial injury, defined according to the Fourth Universal Definition of Myocardial Infarction, was associated with adverse outcomes after acute IS [28]. However, an important methodological difference is that Stengl et al. specifically evaluated dynamic changes in cardiac troponin, whereas our retrospective analysis used the first available and the highest recorded hs-cTnI values obtained during routine clinical care. The timing and frequency of repeat measurements were therefore not standardised. Consequently, our peak hs-cTnI variable should be interpreted as the highest clinically available concentration during hospitalisation, rather than as a protocol-defined serial biomarker trajectory. Prospective studies with predefined sampling intervals are needed to determine whether troponin dynamics provide additional prognostic information beyond individual admission or peak measurements.

A notable finding of our study was the more pronounced hs-cTnI elevation in patients with HS. hs-cTnI elevation was substantially more frequent in HS, and both admission and peak concentrations were higher than in ischaemic ACVD. These findings suggest a greater myocardial injury signal in HS and are consistent with substantial activation of brain–heart pathways following intracranial haemorrhage. Acute haemorrhagic brain injury may result in pronounced sympathetic activation, catecholamine release, autonomic dysregulation, and systemic inflammatory responses, all of which may contribute to myocardial injury. Emerging evidence supports the clinical relevance of this phenomenon. Rosso, Stengl, Scheitz and colleagues demonstrated that acute myocardial injury occurs in spontaneous intracerebral haemorrhage and that dynamic myocardial injury carries prognostic significance. Other studies have similarly reported associations between dynamic troponin elevation and adverse outcomes in intracerebral haemorrhage [29,30,31]. These findings align with our observations of more pronounced myocardial injury in HS, although methodological differences across studies should be considered. Further studies are warranted, as evidence on SHS in HS remains limited.

The pattern of associations with LOS differed from that observed for mortality. In univariable analyses, longer hospitalisation was associated with older age, greater neurological and functional impairment (NIHSS and mRS), and lower LVEF, whereas hs-cTnI and NT-proBNP were not significantly associated with LOS. However, none of the variables in the multivariable models remained significantly associated with LOS. These findings suggest that LOS likely reflects a complex interplay among neurological severity, functional status, cardiovascular reserve, and other clinical or organisational factors not fully captured in the present models. Nevertheless, the univariable association between lower LVEF and longer LOS supports continued consideration of cardiac function within comprehensive stroke assessment, although our data do not establish LVEF as an independent determinant of LOS. This broader perspective is consistent with the integrated management of stroke and associated cardiovascular disease advocated by the ESC Council on Stroke [26].

AF warrants particular consideration, as it is both a major cause of IS and a common cardiovascular comorbidity after stroke. In our cohort, AF was present in approximately one-third of patients, highlighting the substantial overlap between cerebrovascular and cardiac disease. This supports an integrated approach to AF management, consistent with the Atrial Fibrillation Better Care (ABC) pathway proposed by Lip et al.: ‘A’—Avoid stroke with appropriate anticoagulation; ‘B’—Better symptom management; and ‘C’—Cardiovascular and comorbidity optimisation. This concept has since evolved into the AF-CARE (Atrial Fibrillation–Comorbidity management, Avoid stroke and thromboembolism, Reduce symptoms, Evaluation and dynamic reassessment) framework, endorsed by contemporary ESC guidelines, which emphasises comorbidity management, prevention of stroke and thromboembolism, symptom control, and dynamic reassessment [32].

Beyond oral anticoagulation, comprehensive thromboembolic prevention may also include left atrial appendage occlusion for appropriately selected patients. Previous studies, including a systematic review and meta-analysis, have examined percutaneous and surgical left atrial appendage closures and their association with stroke and mortality [33,34]. However, the roles of these strategies specifically in patients with acute SHS and their potential relationships with early post-stroke outcomes remain to be established.

Our observations are clinically consistent with contemporary guidelines emphasising early CV assessment after acute stroke. The 2026 American Heart Association/American Stroke Association (AHA/ASA) guideline supports baseline ECG and cardiac troponin assessment as part of the initial evaluation, while stressing that cardiac investigations should not delay reperfusion treatment [35]. Our findings extend this concept by suggesting that NT-proBNP and assessment of systolic cardiac function may provide complementary prognostic information, although prospective studies are required before routine SHS-specific biomarker algorithms can be recommended.

Environmental exposures may also contribute to the broader CV risk profile of patients presenting with acute stroke. Our previous population-based studies within the PL-PARTICLES and EP-PARTICLES population-wide studies demonstrated associations between air pollution and artificial light at night and CV mortality, AF hospitalisations, and CV morbidity [36,37,38]. These population-level observations provide a rationale for investigating whether environmental exposures may also modify susceptibility to cardiac complications after acute stroke. The present study did not assess environmental exposures; therefore, their potential contributions to post-stroke biomarker profiles and clinical outcomes remain unknown. Prospective studies integrating environmental exposures with neurological, cardiac, and biomarker phenotyping may help further characterise interindividual susceptibility to SHS.

### Limitations

Several limitations should be acknowledged, including the single-centre retrospective design, limited generalisability, inability to establish causality, residual confounding from unmeasured variables, and marked imbalance between groups, with the small HS subgroup underpowered for multivariable analyses. In addition, non-standardised biomarker sampling, incomplete echocardiographic data, and the absence of systematic advanced cardiac imaging may have limited the precision of cardiac phenotyping. Detailed information on pre-stroke antithrombotic therapy, including anticoagulant type and dose and antiplatelet treatment, was incomplete, precluding reliable treatment-stratified analyses.

Despite these limitations, this study has several strengths, including the simultaneous assessment of cardiac biomarkers, neurological severity, and left ventricular systolic function in a real-world acute stroke population. The inclusion of both ischaemic and haemorrhagic cerebrovascular events also provides insight into potential differences in the cardiac response across stroke subtypes.

## 5. Conclusions

SHS represents an important manifestation of cardiac involvement following acute stroke and may contribute to short-term outcomes. Our findings support an integrated neurological and cardiovascular approach to early risk assessment after acute stroke. Future prospective studies should focus on developing and validating SHS-specific risk-stratification strategies integrating dynamic cardiac biomarkers, cardiac function, and neurological status to guide targeted monitoring and management.

## Figures and Tables

**Table 1:** Baseline characteristics of the study group, with cardiac biomarkers, left ventricle ejection fraction (LVEF), neurological status, and in-hospital outcomes in patients with haemorrhagic stroke (HS) and acute ischemic cerebrovascular disease (ACVD).

Variable	Total *n* = 586 (100%)	HS *n* = 26 (4.4%)	Ischaemic ACVD *n* = 560 (95.6%)	*p*-Value
Age, years (median [IQR])	75 (67–84)	73 (66–78)	75 (67–85)	0.18
Sex, male	47.61% (279)	53.85% (14)	47.32% (265)	0.52
AF	33.96% (199)	30.77% (8)	34.11% (191)	0.73
CAS	80.38% (471)	61.54% (16)	81.25% (455)	0.01
HF	29.18% (171)	42.31% (11)	28.57% (160)	0.13
T2DM	27.47% (161)	38.46% (10)	26.96% (151)	0.12
Hypertension	88.05% (516)	88.46% (23)	88.04% (493)	0.95
Hyperlipidaemia	59.90% (351)	53.85% (14)	60.18% (337)	0.52
CKD	10.24% (60)	19.23% (5)	9.82% (55)	0.12
CCS	13.14% (77)	15.38% (4)	13.04% (73)	0.73
Past MI	7.85% (46)	7.69% (2)	7.68% (43)	1
Past IS	12.29% (72)	3.85% (1)	12.68% (71)	0.18
Past TIA	3.58% (21)	0.00% (0)	3.75% (21)	0.32
Cardiac biomarkers and LVEF				
hs-cTnI > 15.6, ng/L	44.5% (266)	73.1% (19)	44.1% (247)	0.004
hs-cTnI > 10 × URL (156 ng/L)	12.6% (74)	19.2% (5)	12.3% (69)	0.3
LVEF, %	54 (47–56)	52.5 (45–55)	54 (47–56)	0.18
1st hs-cTnI, ng/L	10 (10–27.35)	21.1 (10–33.6)	10 (10–27.1)	0.03
Peak hs-cTnI, ng/L	12.9 (10–42.5)	29.8 (12.7–53.0)	12.5 (10–42.5)	0.02
1st NT-proBNP, pg/mL	996.6 (192.7–3240.9)	456.6 (162.4–2888.5)	1017.95 (195.4–3286.9)	0.74
Peak NT-proBNP, pg/mL	1018 (204.6–3286.9)	456.6 (162.4–2888.5)	1045.2 (227.4–3452.5)	0.6
Neurological status				
NIHSS, points	7 (3–14)	8 (1–16)	7 (3–14)	0.93
mRS, points	3 (2–5)	5 (1–5)	3 (2–5)	0.22
In-hospital outcomes				
In-hospital mortality	7.5% (44)	3.9% (1)	7.7% (43)	0.47
Composite in-hospital clinical endpoint *	17.4% (102)	23.1% (6)	17.1% (96)	0.44
In-hospital stay, days	8 (5–10)	16.5 (4–20)	8 (5–9)	<0.001

* Composite in-hospital clinical endpoint defined as MI, hs-cTnI > 10 × URL (156 ng/L), or death. Abbreviations: AF, atrial fibrillation; CAS, carotid artery stenosis; CCS, chronic coronary syndrome; CKD, chronic kidney disease; T2DM, type 2 diabetes mellitus; HF, heart failure; hs-cTnI, high-sensitivity troponin I; IS, ischaemic stroke; LVEF, left ventricle ejection fraction; MI, myocardial infarction; mRS, modified Rankin Scale; NIHSS, National Institutes of Health Stroke Scale; NT-proBNP, N-terminal pro-B-type natriuretic peptide; SD, standard deviation; TIA, transient ischaemic attack; URL, upper reference limit.

**Table 2:** Univariable and multivariate predictors of in-hospital death and length of hospitalisation in patients with stroke.

Predictors of In-Hospital Death for all Stroke Events—Univariable Regression	OR	SE	95% CI	*p*-Value
Age; per 1 year	1.039	0.014	1.011–1.068	0.006
1st hs-cTnI, ng/L; per 1000	1.066	0.024	1.020–1.114	0.004
Peak hs-cTnI, ng/L; per 1000	1.067	0.020	1.003–1.011	0.001
1st NT-proBNP, pg/mL; per 1000	1.111	0.031	1.051–1.172	<0.001
Peak NT-proBNP, pg/mL; per 1000	1.105	0.028	1.051–1.162	<0.001
NIHSS, points	1.101	0.035	1.035–1.172	0.002
mRS, points	1.574	0.217	1.202–2.062	0.001
LVEF upon AS, [%]	0.945	0.017	0.912–0.978	0.001
**Predictors of in-hospital death for all stroke events—multivariable regression—model 1**	**OR**	**SE**	**95% CI**	***p*** **-value**
Age; per 1 year	1.046	0.033	0.984–1.111	0.15
HF prior to AS	0.332	0.256	0.073–1.503	0.15
Peak hs-cTnI; ng/L; per 1000	1.001	0.046	0.914–1.096	0.98
NT-proBNP, pg/mL; per 1000	1.114	0.044	1.032–1.203	0.006
LVEF upon AS, [%]	0.978	0.035	0.912–1.048	0.53
**Predictors of in-hospital death for all stroke events—multivariable regression—model 2**	**OR**	**SE**	**95% CI**	***p*** **-value**
Age; per 1 year	1.023	0.033	0.960–1.089	0.49
HF prior to AS	0.406	0.286	0.102–1.616	0.2
ln Peak hs-cTnI, ng/L	1.236	0.191	0.912–1.674	0.17
ln Peak NT-proBNP, pg/mL	1.866	0.586	1.008–3.452	0.05
LVEF upon AS, [%]	1.006	0.038	0.934–1.085	0.87
**Length of hospitalisation (days)** **—univariable regression**	**β**	**SE**	**95% CI**	***p*** **-value**
Age; per 1 year	0.038	0.016	0.006–0.07	0.02
NIHSS, points	0.096	0.048	0.0008–0.19	0.05
mRS, points	0.6	0.208	0.19–1.01	0.004
1st hs-cTnI, ng/L	−0.00003	0.00006	−0.0001–0.00008	0.6
Peak hs-cTnI, ng/L	−0.00002	0.00005	−0.0001–0.00007	0.7
NT-proBNP, pg/mL	0.00008	0.00007	−0.00006–0.0002	0.2
Peak NT-proBNP, pg/mL	0.00007	0.00006	−0.00005–0.0002	0.3
LVEF upon AS, [%]	−0.116	0.0267	−0.17–0.064	<0.001
**Length of hospitalisation (days)** **—multivariable regression—model 1**	**β**	**SE**	**95% CI**	***p*** **-value**
Age; per 1 year	−0.019	0.031	−0.08–0.043	0.54
Peak hs-cTnI, ng/L	0.00004	0.00009	−0.0001–0.0002	0.62
Peak NT-proBNP, pg/mL	0.00002	0.00008	−0.0001–0.0002	0.79
LVEF upon AS, [%]	−0.094	0.049	−0.19–0.0025	0.056
**Length of hospitalisation (days)** **—multivariable regression—model 2**	**β**	**SE**	**95% CI**	***p*** **-value**
Age; per 1 year	−0.034	0.034	−0.102–0.033	0.32
ln Peak hs-cTnI, ng/L	0.17	0.27	−0.36–0.7	0.53
ln Peak NT-proBNP, pg/mL	0.28	0.35	−0.404–0.97	0.42
LVEF upon AS, [%]	−0.064	0.053	−0.17–0.041	0.23

## Data Availability

The datasets generated and/or analysed during the current study are available from the corresponding author (M.C.) upon reasonable request. Public deposition of the data is restricted due to ethical approval conditions and the protection of participants’ privacy.

## Supplementary Materials

The following supporting information can be downloaded at: https://www.mdpi.com/article/10.3390/jcm15197343/s1, Figure S1. Receiver operating characteristic (ROC) curves for peak cardiac biomarker concentrations in predicting in-hospital mortality. (A) Peak high-sensitivity cardiac troponin I (hs-cTnI), AUC = 0.774. (B) Peak N-terminal pro-B-type natriuretic peptide (NT-proBNP), AUC = 0.742. The diagonal line represents discrimination expected by chance (AUC = 0.50). AUC, area under the receiver operating characteristic curve; hs-cTnI, high-sensitivity cardiac troponin I; NT-proBNP, N-terminal pro-B-type natriuretic peptide.

## Abbreviations

The following abbreviations are used in this manuscript:

ABC	Atrial Fibrillation Better Care
ACVD	Acute cerebrovascular disease
ACS	Acute coronary syndrome
AF	Atrial fibrillation
AF-CARE	Atrial Fibrillation–Comorbidity management, Avoid stroke and thromboembolism, Reduce symptoms, Evaluation and dynamic reassessment
AHA/ASA	American Heart Association/American Stroke Association
ASE	American Society of Echocardiography
CAD	Coronary artery disease
CAS	Carotid artery stenosis
CCS	Chronic coronary syndrome
CI	Confidence interval
CKD	Chronic kidney disease
CMIA	Chemiluminescent microparticle immunoassay
DM	Diabetes mellitus
EACVI	European Association of Cardiovascular Imaging
ECG	Electrocardiography
ESC	European Society of Cardiology
HF	Heart failure
HS	Haemorrhagic stroke
hs-cTnI	High-sensitivity cardiac troponin I
HT	Hypertension
ICU	Intensive care unit
IQR	Interquartile range
IS	Ischaemic stroke
LOS	Length of hospital stay
LVEF	Left ventricular ejection fraction
MI	Myocardial infarction
mRS	Modified Rankin Scale
NASCET	North American Symptomatic Carotid Endarterectomy Trial
NIHSS	National Institutes of Health Stroke Scale
NT-proBNP	N-terminal pro-B-type natriuretic peptide
OR	Odds ratio
SD	Standard deviation
SE	Standard error
SHS	Stroke–heart syndrome
TIA	Transient ischaemic attack
TOAST	Trial of Org 10172 in Acute Stroke Treatment
URL	Upper reference limit
