# Supplementary material for: Distinct Determinants of Hospitalisation and In-Hospital Mortality After Acute Stroke: Insights into Stroke–Heart Syndrome (SHS-PARTICLES Study)
Source: J Clin Med. 2026 Sep 22;15(19):7343. doi: 10.3390/jcm15197343 (PMC13666459; doi:10.3390/jcm15197343)
Supplement: Supplementary file 1 [file jcm-15-07343-s001.zip › jcm-4519366-supplementary.pdf]

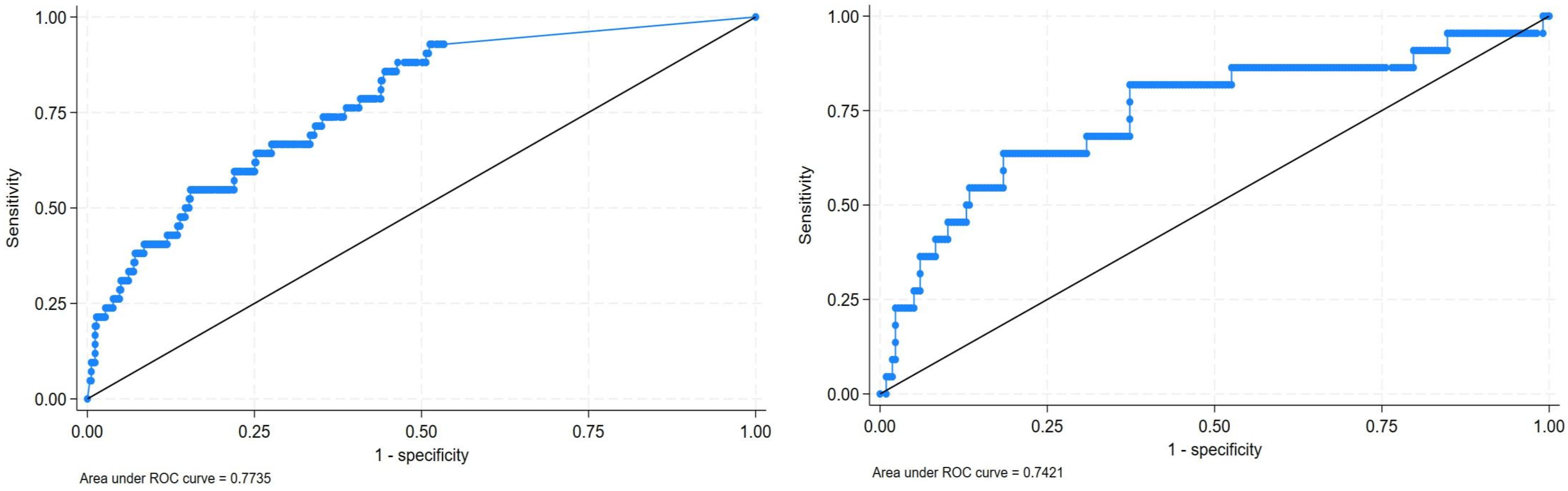

(A) **(B)**

Figure S1. Receiver operating characteristic (ROC) curves for peak cardiac biomarker concentrations in predicting in-hospital mortality. (A) Peak high-sensitivity cardiac troponin I (hs-cTnI), AUC = 0.774. (B) Peak N-terminal pro-B-type natriuretic peptide (NT-proBNP), AUC = 0.742. The diagonal line represents discrimination expected by chance (AUC = 0.50). AUC, area under the receiver operating characteristic curve; hs-cTnI, high-sensitivity cardiac troponin I; NT-proBNP, N-terminal pro-B-type natriuretic peptide.
